# Foundations and Strategic Vision of the Canadian Translational Geroscience Network

**DOI:** 10.1093/gerona/glaf111

**Published:** 2025-05-19

**Authors:** Guy Hajj-Boutros, Andréa Faust, John Muscedere, Perry Kim, Gilles Gouspillou, Lea Harrington, James L Kirkland, George A Kuchel, Jeremy Van Raamsdonk, R Jane Rylett, Chantal Autexier, Louis R Lapierre, Michael Kobor, Mohammad Auais, Ann Beliën, Jeroen Aerssens, George Sutphin, Kenneth Rockwood, Alexandra Papaioannou, Marc Sim, Jamie Justice, Nancy Mayo, Gustavo Duque

**Affiliations:** Department of Medicine, McGill University and Research Institute of the McGill University Health Centre, Montréal, Québec, Canada; Department of Medicine, McGill University and Research Institute of the McGill University Health Centre, Montréal, Québec, Canada; Simone & Edouard Schouela RUISSS McGill Centre of Excellence for Sustainable Health of Seniors (Schouela CEDurable), Montréal, Québec, Canada; Canadian Frailty Network, Kingston, Ontario, Canada; Department of Critical Care Medicine, Queen’s University, Kingston, Ontario, Canada; Canadian Frailty Network, Kingston, Ontario, Canada; Département des Sciences de l’Activité Physique, Faculté des Sciences, Université du Québec à Montréal, Montréal, Québec, Canada; Department of Biochemistry, University of Toronto, Toronto, Ontario, Canada; Center for Advanced Gerotherapeutics, Cedars-Sinai Medical Center, Los Angeles, California, USA; UConn Center on Aging, University of Connecticut School of Medicine, UConn Health, Farmington, Connecticut, USA; Department of Neurology and Neurosurgery, McGill University, Metabolic Disorders and Complications (MeDiC) Program, RI-MUHC, Montréal, Québec, Canada; Department of Physiology and Pharmacology, Western University, London, Ontario, Canada; Department of Medicine, McGill University, Montréal, Québec, Canada; Departement de chimie et biochimie, Université de Moncton, Moncton, Nouveau-Brunswick, Canada; Department of Medical Genetics, University of British Columbia, Vancouver, British Columbia, Canada; Department of Rehabilitation Sciences, Qatar University, Doha, Qatar; School of Rehabilitation Therapy, Queen’s University, Kingston, Ontario, Canada; Rejuvenate Biomed, Diepenbeek, Belgium; Rejuvenate Biomed, Diepenbeek, Belgium; Department of Molecular and Cellular Biology, University of Arizona, Tucson, Arizona, USA; Department of Medicine, Dalhousie University, Halifax, Canada; Departments of Medicine and HEI, McMaster University, Hamilton, Ontario, Canada; Nutrition and Health Innovation Research Institute, Edith Cowan University, Perth, Australia; XPRIZE Healthspan, Culver City, California, USA; School of Physical and Occupational Therapy, McGill University, Montréal, Québec, Canada; Department of Medicine, McGill University and Research Institute of the McGill University Health Centre, Montréal, Québec, Canada; Simone & Edouard Schouela RUISSS McGill Centre of Excellence for Sustainable Health of Seniors (Schouela CEDurable), Montréal, Québec, Canada; (Biological Sciences Section)

**Keywords:** Aging, Clinical trials, Geroscience, Nutraceuticals, Translational research

## Abstract

Geroscience is an emerging interdisciplinary field that explores the biological connections between aging and the development of chronic diseases, with the ultimate goal of identifying interventions to extend healthspan and delay age-related conditions. Recognizing the growing importance of this field, the Canadian Translational Geroscience Network (geroscience.ca) was officially launched during a conference held in Montreal on September 5–6, 2024. Building on the momentum of successful Geroscience meetings in Toronto and Montreal in 2023, this milestone event marked a transformative step forward for geroscience in Canada. This event brought together key stakeholders, including the Canadian Frailty Network (CFN), the Canadian Institutes of Health Research Institute of Aging (CIHR-IA), the Réseau Québécois de Recherche sur le Vieillissement (RQRV), the Simone & Edouard Schouela RUISSS McGill Centre of Excellence for Sustainable Health of Seniors (Schouela CEDurable), the Division of Geriatric Medicine at McGill University, and the Department of Biochemistry at the University of Toronto. Additionally, a broad coalition of geriatricians, healthcare professionals, and researchers convened to discuss and advance the field of geroscience in Canada. The 2-day conference focused on creating a multidisciplinary community to address the challenges of an aging population, emphasizing the importance of funding, national and international collaboration, and training the next generation of researchers and clinicians. Workshops and presentations showcased a range of innovative research, from cellular studies to clinical trials, aimed at understanding and treating age-related diseases. Key discussions highlighted the critical role of partnerships among research institutions, healthcare systems, and biotech companies in translating research findings into practical interventions. The Canadian Translational Geroscience Network’s strategic objectives focus on expanding funding opportunities for geroscience, developing specialized training programs, and increasing membership to cultivate a diverse, multidisciplinary, and collaborative network. This network aims to include students, basic and clinical researchers, citizens, government entities, and organizations or professionals interested in advancing the geroscience field. With a clear roadmap for future growth, the Canadian Translational Geroscience Network aims to position Canada at the forefront of geroscience, fostering evidence-based innovation that improves the health and quality of life for aging populations.

In recent decades, scientific inquiry into the relationship between the biology of aging and the development of chronic diseases has led to the emergence of geroscience. This field focuses on understanding the fundamental mechanisms of aging and leveraging that knowledge to create innovative therapies and preventive measures. By targeting the core biological drivers of aging, geroscience aims to reduce the collective burden of age-related diseases and enhance the quality and duration of healthy life (1). The growing importance of geroscience becomes particularly evident in the context of Canada’s aging population and the rising number of frail older adults. By 2036, population projections estimate that the number of adults aged 65 years and above could reach between 9.9 and 10.9 million (upward of 25% of the total population), and the number of people aged 80 and older, who are most likely more vulnerable, could more than double to 3.3 million (2). This demographic shift underscores the urgent need to promote healthy aging and mitigate the risks of diseases commonly associated with advancing age. Achieving this goal is not only vital for improving the quality of life for older adults but also for reducing the strain on healthcare systems.

Despite the increasing recognition of geroscience, many researchers and healthcare professionals unknowingly contribute to this field through studies addressing aging-related mechanisms without explicitly identifying their work as geroscience. This lack of alignment highlights the need for a unified approach to foster collaboration, share knowledge, and translate research findings into actionable interventions. Strengthening such connections will be essential to harness the full potential of geroscience in addressing the challenges of an aging population.

Building on the success and momentum of the 2 Geroscience meetings held in Toronto and Montreal in 2023, along with the publication that underscored the pressing need for a unified Geroscience Network in Canada (3), the Canadian Frailty Network, the Canadian Institutes of Health Research (CIHR), the Réseau Québécois de Recherche sur le Vieillissement (RQRV), and a broad coalition of geriatricians, physicians, healthcare professionals, and researchers came together on September 5–6, 2024, in Montreal to officially launch the Canadian Translational Geroscience Network (CTGN, geroscience.ca). This momentous gathering represented a significant step forward in advancing the field of geroscience in Canada, providing a formal platform for collaboration, innovation, and the translation of substantial research into practical health interventions for aging populations.

The discussions throughout the 2-day event centered on the pivotal role of the newly established network, emphasizing the importance of creating a multidisciplinary community to address the growing challenges an aging population faces. Key topics included the identification and promotion of new geroscience funding opportunities, strategies to enhance national and international geroscience research collaboration, and the need to actively engage a wide range of scientists, clinicians, healthcare professionals, and older adults’ voices in geroscience research. Special attention was given to the importance of training the next generation of researchers and clinicians in the field to ensure sustained progress in geroscience across Canada and internationally.

Workshops and presentations showcased a broad spectrum of ongoing geroscience research in Canada and internationally, ranging from cellular and molecular studies to clinical trials. These sessions highlighted innovative approaches to preventing and treating age-related diseases by targeting the hallmarks of aging, such as targeting cellular senescence, enhancing mitochondrial and lysosomal function, and understanding the molecular pathways that reduce/prevent chronic disease and contribute to healthy aging. Equally important were discussions on how to translate these findings into real-world healthcare solutions, emphasizing the need for practical and sustainable evidence-based interventions that can be implemented to improve the quality of life for older adults.

The conference also explored the roles of key partners, including governmental bodies, research institutions, healthcare systems, and private-sector stakeholders, in shaping the future of geroscience in Canada. These partnerships will be critical in advancing a cohesive national strategy that fosters collaboration and ensures the network’s sustainability. Discussions emphasized the need for inclusive, multisector partnerships to ensure the network remains flexible and responsive to the evolving landscape of aging and geroscience research and healthcare.

In this article, we provide a comprehensive overview of the key discussions that took place during the conference, highlight the most significant ongoing geroscience research efforts both in Canada and globally, and outline the strategic objectives for the CTGN. By establishing a clear roadmap for the future, we aim to position Canada at the forefront of geroscience innovation, ensuring that our healthcare systems, policies, and scientific research are well-prepared to meet the challenges and opportunities of an aging population.

## Workshop and Ongoing Research Discussed During the Conference

The first day of the conference featured several workshops dedicated to advancing both fundamental research and clinical trials in geroscience. The sessions started with an in-depth discussion on the hallmarks of aging, bridging the gap from bench to bedside. Experts from institutions such as McGill University, Université de Moncton, and the University of British Columbia shared their insights on translating cellular and molecular findings into clinical settings (4,5).

A special workshop focused on designing geroscience clinical trials was led by distinguished researchers from Queen’s University, the University of Connecticut, and Cedars-Sinai (6–9). This session covered essential components of clinical trial design, emphasizing strategies for achieving successful study applications in the context of aging research and geroscience. This workshop was particularly beneficial for early career investigators, providing them with crucial guidance on navigating the complexities of clinical trial design in geroscience. Lastly, a separate workshop focused on bringing scientific discoveries to real life. This session explored topics like intellectual property, commercialization, and the practical implementation of research findings. Presenters from McGill University and the biotech company Rejuvenate Biomed (Belgium) highlighted the pathways to transforming innovative research into tangible healthcare solutions, aiming to make these interventions accessible to aging populations.

On the second day, following the official launch of the CTGN, leading Canadian and international experts presented their ongoing research. Discussions included strategies for collaboration and data-sharing, focusing on the use of Biobanks and shared databases to advance geroscience research. These presentations highlighted the critical need to integrate clinical insights with translational research to address the complex challenges associated with aging, such as frailty (10,11). The role of advanced technologies also emerged as a key topic within the field of geroscience (12). Additionally, exercise and nutrition were recurring themes, emphasizing the critical role of physical activity in promoting overall health and maintaining functional capacity in older persons (13–16). Evidence further suggests that regular physical activity is among the most significant factors contributing to healthy aging (17), warranting special attention from all Canadians as a geroscience intervention that can be implemented today to improve health and well-being.

However, it is essential to approach lifestyle interventions, including exercise and nutrition, with a nuanced perspective. While physical activity has robust evidence supporting its benefits, many other lifestyle interventions, such as ensuring appropriate sleep and addressing sleep disorders, also hold significant promise. For some interventions, the existing evidence is primarily associative or retrospective, with a limited number of carefully controlled prospective clinical trials. This underscores the need for further research to determine the relative effectiveness of various approaches.

Rather than prematurely endorsing one specific lifestyle modification, natural product, repurposed drug, new chemical entity, medical device, or other intervention as superior, a broader focus on combinations and sequencing of interventions tailored to individual needs may be more beneficial. Personalized strategies that integrate multiple interventions, adjusted to an individual’s unique biology, preferences, and circumstances, may yield more significant outcomes than any single approach. This perspective highlights the importance of advancing geroscience research to explore these complex interactions and optimize interventions for healthy aging.

The agenda also featured a series of short presentations by students and early career investigators, showcasing a diverse range of topics, from the metabolic effects of bed rest (18) to artificial intelligence-driven approaches for computational phenotyping (19). These presentations provided a platform for emerging geroscience researchers to share their innovative work and receive feedback from established experts, fostering an environment of mentorship and collaborative growth.

Throughout the conference, a recurring theme was the emphasis on multi- and interdisciplinary collaboration and the need to engage various stakeholders in the field of geroscience. Discussions highlighted the importance of partnerships among governmental bodies, academic institutions, healthcare systems, and industry players to develop a cohesive national strategy for advancing aging and geroscience research in Canada.

This comprehensive approach aims to accelerate the translation of geroscience research findings into practical interventions that improve the health and quality of life of older adults. It ultimately positions the CTGN at the forefront of geroscience innovation.

## Strategic Objectives and Structure of the CTGN

During the conference, several strategic objectives and structural elements of the CTGN were discussed, with a clear emphasis on expanding its mandate to include a robust educational component. This focus on training the next generation of researchers and clinicians was highlighted as a crucial step in ensuring sustained progress in geroscience. The objective to advocate for joint practices, standards, and protocols was also emphasized, along with the need to develop an inventory of research sites capable of hosting and participating in clinical studies, particularly in the important fields of gerotherapeutics, nutraceuticals, and medical devices. Recognizing the value of incorporating older persons’ perspectives, the discussions underscored the importance of exposing trainees to the voices and experiences of this population, thereby enhancing the relevance and impact of geroscience interventions. The network aims to establish intervention testing programs to support career development and create opportunities for early career researchers and trainees, such as positions encouraging active participation and motivation.

In terms of membership, the network plans to broaden its inclusivity by integrating community members and offering roles specifically for trainees through connections with associations. Expanding the membership criteria to include individuals involved in research, education, or implementation reflects the network’s commitment to a multidisciplinary approach. A representative from each province and territory will be included to ensure a national perspective, with clearly defined roles for each position. Coordinators will handle communications and social media, while executive members will oversee specific areas like research development and trainee support. To strengthen the network’s infrastructure, there was a call for expanding collaborations and locating the annual meeting in different provinces. The University of Toronto was the suggested institution to host the 2025 annual meeting. Membership fees were also discussed, with a consensus on implementing a fee structure that varies depending on the member’s role, such as reduced rates for trainees and remote territories. It was suggested that membership fees could be integrated into scientific meeting costs and grant budgets to support the network’s sustainability. A proposal to automatically grant membership upon event attendance was also considered. While the majority voted in favor of a membership fee, it was agreed that a new committee should make the final decision, potentially involving another survey to gauge member preferences. Finally, creating roles for recently graduated professionals from geroscience-related fields was seen as a priority, highlighting the network’s commitment to nurturing future leaders in this evolving area of research.

## Funding Landscape for Geroscience Research

One of the central themes discussed at this year’s conference was the critical role of funding agencies in advancing geroscience research. Effective coordination with these bodies is essential to transforming the insights gained from fundamental and clinical research into impactful health interventions for aging populations. In Canada, the Canadian Institutes of Health Research (CIHR) has been a key player in supporting studies that bridge biomedical and clinical sciences. The CIHR comprises 13 Institutes, each working toward advancing scientific excellence and generating knowledge that benefits the health of Canadians within their individual mandate areas and in collaboration. Among these, the Canadian Institutes of Health Research—Institute of Aging (CIHR-IA) takes a lead role in understanding the biological, clinical, and social aspects of aging, aiming to improve the health and quality of life of older persons. The continuous support from CIHR-IA for funding studies in this field is crucial and will help advance geroscience research in Canada.

The Canadian Frailty Network (CFN), a cofounder of the CTGN, was a significant contributor to the discussions, emphasizing its commitment to tackling the challenges of aging and frailty through translational research (20). The CFN’s initiatives focus on preventing aging-related health decline and supporting innovative approaches in geroscience that hold promise for enhancing the well-being of older persons. Their role as a national organization positions them as a crucial force in shaping the trajectory of geroscience research and its implementation across the country. The CFN is dedicated to supporting geroscience research in Canada. Similarly, the Fonds de Recherche du Québec—Santé (FRQS) was highlighted for its pivotal support in driving aging research in Quebec. With a focus on fostering collaborative efforts and supporting a spectrum of projects from fundamental discoveries to clinical applications, FRQS plays a vital role in advancing the province’s contributions to the broader field of geroscience.

The conference underscored the importance of these agencies not only in funding individual projects but also in fostering collaborative efforts that drive the translation of research findings into real-world clinical applications. By investing in interdisciplinary and cross-sector initiatives, these organizations help bridge the gap between scientific discovery and patient care, ultimately enhancing the quality of life for Canada’s aging population.

A notable point of discussion was the need for enhanced international collaboration, particularly with the National Institute on Aging (NIA) in the United States. The CIHR-IA, CFN, and FRQS have actively engaged in initiatives that align Canadian research priorities with those of the NIA, facilitating a robust exchange of expertise, resources, and data. This transnational partnership aims to leverage diverse populations and larger sample sizes to produce more comprehensive insights into the biology and treatment of aging. Participants at the conference emphasized that this international synergy not only strengthens the research infrastructure but also accelerates the pace of innovations in geroscience.

An exciting new development gaining attention in Canada is the announcement of an XPrize for Healthspan. This ambitious initiative aims to incentivize breakthroughs in science and technology that extend healthspan, the period of life spent in good health, by addressing the fundamental biological and societal challenges of aging. By encouraging innovative solutions, the prize seeks to catalyze transformative advancements in geroscience, healthcare, and public health. The XPrize for Healthspan, with a remarkable award of $100 million USD, represents one of the most significant investments in aging research and intervention development to date. The initiative is expected to draw participation from interdisciplinary teams worldwide, including researchers, biotech companies, and policymakers, fostering a global race to unlock new strategies for enhancing the quality of life for older adults.

Beyond the monetary value, the XPrize serves as a symbol of the growing recognition of aging as a critical area for innovation. It emphasizes the need for bold, scalable solutions to address the increasing healthcare demands of aging populations. With Canada playing a central role in this groundbreaking initiative to improve the healthcare system and address the challenges of an aging population, the prize has the potential to establish the country as a global leader in geroscience, while fostering international collaboration and attracting investment aimed at promoting longer, healthier lives for all. This initiative aligns with the overarching goals of the geroscience community, underscoring the importance of promoting healthspan alongside lifespan and demonstrating the power of incentive-driven research to tackle some of society’s most pressing challenges.

As the geroscience community moves forward, fostering a global network of funding and research initiatives will be crucial. The shared vision of national and international agencies will drive the development of groundbreaking interventions to address the complexities of aging, with a particular focus on improving health outcomes and extending healthy years of life for older persons. The conference called for a continued commitment to these collaborations, emphasizing that the collective efforts of these funding bodies will be essential to maintaining momentum and ensuring the sustainable growth of the field. The CTGN could position itself as the leading organization recognized by funding bodies for its pivotal role in supporting researchers to undertake projects with tangible, real-world impact. By fostering a seamless connection between bench-to-bedside research and broader community applications, CTGN bridges the gap between scientific discovery and practical implementation, ensuring that innovations in geroscience translate into meaningful improvements in health and well-being for aging populations. This support aims to meet the needs of scientists and increase the feasibility of smaller-scale projects, providing a valuable platform for innovative ideas and helping early career investigators launch their careers and thrive in the field of geroscience.

## Education and the Development of Geroscience Programs

Education was a central theme at this year’s conference, strongly emphasizing cultivating the next generation of geroscience researchers and clinicians. A key initiative proposed was the creation of specialized programs, including a master’s degree, to equip early-career researchers with the skills to address the biological, clinical, and social aspects of aging. This program would integrate the best research and practical applications, preparing graduates to lead innovative projects to prevent and treat age-related conditions.

Additionally, the CTGN is committed to incorporating geroscience principles into existing graduate and postgraduate courses across various institutions, ensuring that these concepts are widely accessible to students from diverse academic backgrounds. The network aims to foster partnerships with universities and educational institutions to expand these offerings, ultimately building a skilled workforce capable of advancing geroscience research and translating scientific findings into real-world interventions.

These educational initiatives reflect the network’s long-term vision of empowering future leaders in geroscience and providing them with the tools to navigate the rapidly evolving landscape of aging research. By investing in education and creating structured learning opportunities, the CTGN seeks to establish a solid foundation for sustained innovation and progress in the field, ensuring that Canada remains at the forefront of geroscience globally.

## Opportunities for Undergraduate and Graduate Students in Geroscience

Engaging students at both the undergraduate and graduate levels is crucial for building a robust pipeline of future researchers in the field of geroscience. The CTGN aims to provide a supportive platform that encourages students to start their journey in geroscience early in their academic careers. By leveraging various funding opportunities, educational programs, and professional networks, students can gain valuable experience and make significant contributions to the field from the outset.

For undergraduate students interested in exploring geroscience, there are numerous opportunities to gain hands-on research experience. One notable opportunity is the CIHR Undergraduate Summer Internship Grants program, which provides funding for students to work on research projects during the summer months. This program allows students to immerse themselves in geroscience research, gaining practical skills in laboratory techniques, data analysis, and critical thinking, all under the mentorship of leading researchers in the field. Participating in such internships not only enhances students’ academic and research capabilities but also lays a strong foundation for their future careers in aging research and related disciplines.

### Support from Research Networks: RQRV, CIHR, and CFN

Beyond summer internships, students and early-career researchers can also benefit from the support of established research networks like the RQRV and the CIHR. These organizations offer awards and grants that enable students to attend national and international conferences, where they can present their research, network with experts, and stay informed about the latest developments in geroscience. These travel awards are vital in facilitating knowledge exchange and fostering collaborations across different institutions and countries, enriching the academic experience for young researchers and broadening their professional horizons.

### CIHR-IA Summer Program on Aging for Graduate Students and Postdoctoral Fellows

For PhD students and postdoctoral fellows, the CIHR-IA Summer Program on Aging provides an exceptional opportunity to deepen their understanding of aging-related research. This intensive program is designed to equip participants with advanced knowledge in the field of geroscience, offering a multidisciplinary curriculum that covers topics ranging from the biological mechanisms of aging to the development of interventions that promote healthy aging. The program also emphasizes career development, providing networking opportunities with established scientists, funding agencies, and other stakeholders in the geroscience community. This program is a unique chance for emerging researchers to gain insights into the latest research, engage in meaningful discussions, and build valuable connections through networking opportunities to propel their careers forward both locally and internationally.

### Building a Future in Geroscience

The CTGN is dedicated to supporting and guiding students as they embark on their journey in geroscience. By connecting students with funding opportunities, educational programs, and professional networks, the CTGN aims to inspire the next generation of scientists and clinicians to pursue innovative research addressing aging challenges. With the proper support, undergraduate and graduate students, as well as postdoctoral fellows, can make meaningful contributions to the field, advancing our understanding of aging and ultimately improving the health and quality of life for older persons.

These initiatives are integral to creating a vibrant and dynamic community of geroscience researchers in Canada. By nurturing emerging talent and providing pathways to career development, the CTGN ensures that the field of geroscience will continue to thrive, driven by a new generation of motivated and skilled professionals dedicated to addressing the complexities of aging.

## Future Steps

Moving forward, the CTGN aims to build on the momentum generated at this year’s conference by focusing on 3 key areas: funding, education, and membership expansion. The network will leverage upcoming funding opportunities to support a broader range of geroscience research initiatives. This funding will target innovative projects and help early-career investigators bring novel ideas to fruition, strengthening the overall impact of geroscience studies in Canada.

On the educational front, the CTGN is committed to launching specialized training programs, including a master’s degree in geroscience, designed to equip emerging researchers with the expertise needed to lead advancements in the field. By partnering with universities and educational institutions across the country, the network aims to embed geroscience principles into both existing courses and newly developed curricula, ensuring that future professionals are well-prepared to tackle the challenges of aging.

**Figure 1.**
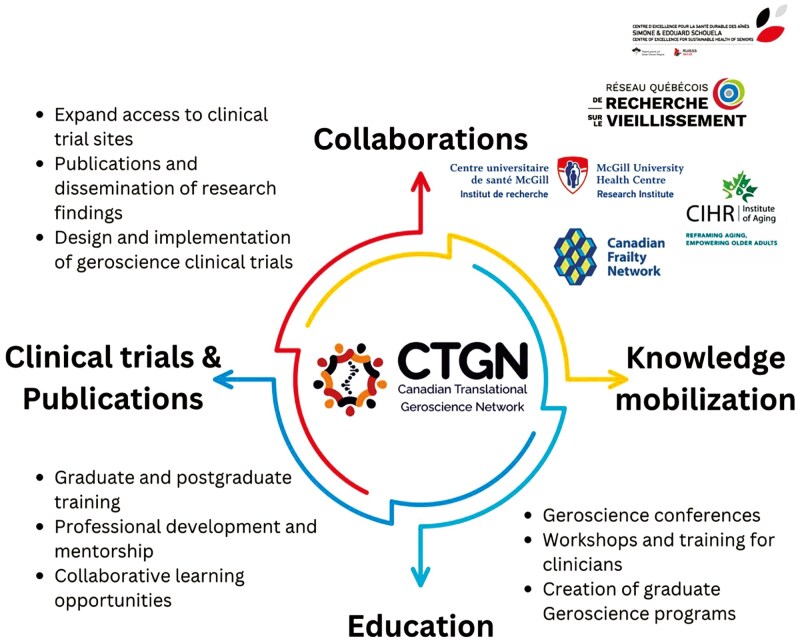
Framework of the Canadian Translational Geroscience Network: integrating clinical research, education, knowledge mobilization, and collaborative partnerships in geroscience.

Expanding the CTGN’s membership remains a top priority, with plans to include a broader and more diverse group of stakeholders from all provinces and territories. By enhancing its network of researchers, clinicians, policymakers, and industry partners, the CTGN seeks to create a vibrant community that fosters collaboration and drives forward interdisciplinary initiatives in aging research (Figure 1). Registration to become a member of the CTGN can be obtained by visiting the following link: https://www.geroscience.ca/.

## Conclusion

The launch of the CTGN has established a strong foundation for collaborative innovation in aging and geroscience research. With its strategic focus on securing funding, advancing education, and expanding its multidisciplinary membership, the CTGN is well-positioned to drive transformative research that addresses the complexities of aging. The network aims to translate discoveries into practical health interventions through a comprehensive pipeline, starting with ideas cocreated with consumers and the community, progressing to evidence generation, and culminating in integration and implementation within the broader community. This end-to-end approach is essential for advancing geroscience and ensuring its impact on improving healthspan and quality of life. As the network grows, bringing together experts from different scientific disciplines across Canada and beyond, it aims to strengthen partnerships and support early career researchers, ultimately positioning Canada as a leader in geroscience. With a clear vision and a commitment to inclusive, multidisciplinary approaches, the CTGN will play a pivotal role in advancing our understanding of aging, developing new geroscience interventions, and ultimately improving the quality of life for older adults.
